# Attention Network Test in adults with ADHD - the impact of affective fluctuations

**DOI:** 10.1186/1744-9081-7-27

**Published:** 2011-07-27

**Authors:** Astri J Lundervold, Steinunn Adolfsdottir, Helene Halleland, Anne Halmøy, Kerstin Plessen, Jan Haavik

**Affiliations:** 1 Department of Biological and Medical Psychology, University of Bergen, Bergen, Norway; 2 Uni Research, Regional Centre for Child and Adolescent Mental Health, Bergen, Norway; 3 Solli Hospital, Bergen, Norway; 4 Department of Biomedicine, University of Bergen, Bergen, Norway; 5 Department of Psychiatry, Haukeland University Hospital, Bergen, Norway; 6 Mental Health Center for Child and Adolescent Psychiatry at Bispebjerg, Capital Region Psychiatry, Copenhagen, Denmark; 7 Department of Neurology, Psychiatry and Sensory Sciences, University of Copenhagen, Copenhagen, Denmark; 8 K.G. Jebsen Center for Research on Neuropsychiatric disorders, Bergen, Norway

## Abstract

**Background:**

The Attention Network Test (ANT) generates measures of different aspects of attention/executive function. In the present study we investigated whether adults with ADHD performed different from controls on measures of accuracy, variability and vigilance as well as the control network. Secondly, we studied subgroups of adults with ADHD, expecting impairment on measures of the alerting and control networks in a subgroup with additional symptoms of affective fluctuations.

**Methods:**

A group of 114 adults (ADHD *n *= 58; controls *n *= 56) performed the ANT and completed the Adult ADHD Rating Scale (ASRS) and the Mood Disorder Questionnaire (MDQ). The latter was used to define affective fluctuations.

**Results:**

The sex distribution was similar in the two groups, but the ADHD group was significantly older (*p *= .005) and their score on a test of intellectual function (WASI) significantly lower than in the control group (*p *= .007). The two groups were not significantly different on measures of the three attention networks, but the ADHD group was generally less accurate (*p *= .001) and showed a higher variability through the task (*p *= .033).

The significance was only retained for the accuracy measure when age and IQ scores were controlled for. Within the ADHD group, individuals reporting affective fluctuations (*n *= 22) were slower (*p *= .015) and obtained a lower score on the alerting network (*p *= .018) and a higher score on the conflict network (*p *= .023) than those without these symptoms. The significance was retained for the alerting network (*p *= .011), but not the conflict network (*p *= .061) when we controlled for the total ASRS and IQ scores.

**Discussion:**

Adults with ADHD were characterized by impairment on accuracy and variability measures calculated from the ANT. Within the ADHD group, adults reporting affective fluctuations seemed to be more alert (i.e., less impacted by alerting cues), but slower and more distracted by conflicting stimuli than the subgroup without such fluctuations. The results suggest that the two ADHD subgroups are characterized by distinct patterns of attentional problems, and that the symptoms assessed by MDQ contribute to the cognitive heterogeneity characterizing groups of individuals with ADHD.

## Background

Attention-deficit/hyperactivity disorder (ADHD) is a neuropsychiatric disorder characterized by motor restlessness and symptoms of impulsivity and inattention. The prevalence in the child population is estimated to be about 5%, and the disorder frequently persists into adulthood [1-4], with symptoms of inattention rather than impulsivity/hyperactivity as the main persistent symptoms [5,6].

Neuropsychological studies have related changes in neural networks involving the frontal lobe to impairment on tests of attention, primarily those defined within the concept of executive function (EF) [7,8]. The importance of EF is emphasized by the fact that impairment in childhood tends to increase into adulthood [9], is associated with severity of ADHD symptoms [10] and overall cognitive and everyday functioning [11]. However, it is well documented that not all individuals with ADHD show impairment on all core tests of EF [12,13], and multiple pathway models have been developed to link different aspects of EF to neurobiology [14,15]. Furthermore, not all impairments associated with ADHD can be explained within the concept of EF [16]. Alterations in more basic perceptual processing [17,18], activation [19] and tempo of information processing [20,21] are reported to influence everyday functioning of individuals with ADHD. A cognitive model of ADHD should therefore describe and operationalize different levels of information processing, their interactions and neurobiological substrates [15].

Posner and colleagues have presented one such model [22]. Their Attention Network Model defines an alerting or vigilance network, an orientation network and an executive or conflict network. The alerting network maintains a high sensitivity to incoming stimuli, the orienting network is involved in selection of information from sensory input, whereas the control network is involved in resolving conflicts between thoughts, feelings and responses [22]. These networks have been associated with different anatomical locations, neurotransmitter systems and genetic markers [23,24], and the model has inspired studies of attention deficits in ADHD as well as in other neuropsychiatric disorders (see [25]).

The Attention Network Model has been used to develop test paradigms that have become popular during the last decades. Fan and collaborators developed the Attention Network Test (ANT) [23,26], and presented updated versions on the Internet. The ANT has recently been used to study cognitive characteristics of individuals with ADHD. A deviant activation pattern in all three networks has been found in fMRI studies of children with ADHD, but impairment was only found on the control network when the test was administrated according to standard procedure outside the scanner [27,28]. A recent Norwegian population-based study could not confirm impairment on any measure of the attention networks in a group of primary school children with ADHD, but a more detailed analysis showed that the children in general performed less accurate and more variable throughout the task than controls [29]. These characteristics were confirmed in an ANT study of college students (age 18-30 yrs) with a combined type of ADHD [30].

The impact of ADHD symptoms on cognitive function is well documented (e.g., [20,31]). Recent studies have also shown that symptoms associated with affective disorders are crucial to understand characteristics of cognition in children [32] and adults [8] with ADHD. Murphy and Barkley illustrated a close association between what they referred to as emotional regulation and metacognition [8], and a longitudinal study emphasized the predictive value of such symptoms on future cognitive and everyday functioning [33]. Other studies have shown that symptoms associated with affective disorders are related to impairment of EF and motivation [34-36], and that children with ADHD and affective disorder (i.e., anxiety) are cognitively distinct from and more impaired than individuals in either condition on measures defined within the concept of EF [32,37]. This may be explained by the heightened arousal characterizing individuals with affective disorders [38], and that this arousal contributes to cognitive impairment through its effect on EF [39]. Due to the high frequency of affective symptoms in adults with ADHD [40], these results motivate further studies using the ANT to investigate characteristics of alerting and control networks.

The aim of the present study was twofold. First, we investigated ANT results in a group of adults with ADHD and a control group from the general population. From earlier studies we expected the ADHD group to show impairment on the ANT measure of the control network, as well as on measures of accuracy, vigilance and variability. Secondly, we investigated ANT results in an ADHD subgroup reporting affective fluctuations. From earlier studies we expected that high arousal and EF impairment in this subgroup would influence their results on the alerting and control networks. Finally, we asked if the results would change when we controlled for ADHD symptoms and intellectual function.

## Methods

The present study included a subset of adults participating in a Norwegian national study of adults with ADHD.

### Participants in the national study

The adults with ADHD were recruited from a national registry of adults diagnosed with ADHD in Norway from 1997 to May 2005. Three national expert committees for ADHD/Hyperkinetic disorders were responsible for the diagnostic assessment, based on information from clinical records provided by the referring clinicians. The records included information collected and evaluated according to the official diagnostic system in Norway, the ICD-10. However, allowance was made for the inattentive subtype in DSM-IV to be sufficient for the diagnosis, so that the assessment also could be comparable with the DSM-IV. A total of 1700 invitation letters were sent from 2005 to 2007, mainly targeting individuals referred after year 2000. Adults with ADHD were also referred directly from Norwegian psychiatrists or psychologists to include individuals diagnosed later than May 2005. These adults were assessed by specialists in clinical psychiatry or psychology according to the national guidelines based on the criteria used by the national expert committees, but without their mandatory evaluation.

The control group was recruited through a random selection from the Norwegian population, using the database of The Medical Birth Registry of Norway (MBRN), which includes all Norwegians born after January 1st 1967. Invitation letters were sent to a randomly selected sample of 2963 individuals who were between 18-40 years old. In addition, a subsample was recruited by different advertisements. The control group was not screened for ADHD before entering the study. However, the prevalence in the population was expected to be low, as the estimate among Norwegian primary school children has been reported to be only 1.7 percent [41]. All participants completed a set of questionnaires (see below for more details). The project was approved by the Regional Committee for Medical Research Ethics of Western Norway and the Norwegian Social Science Data Services (NSD).

### Participants in the present study

We invited randomly selected participants from the main study, living geographically close to the city of Bergen, to take part in a neuropsychological examination including the Attention Network Test and two subtests from the Wechsler Abbreviated Scale of Intelligence (WASI) [42]. The ADHD group comprised 32 females and 26 males, and the control group 34 females and 22 males. Fifty-nine percent of the adults with ADHD used medication related to ADHD, of whom 80 percent used methylphenidate (Ritalin or Concerta). They were asked not to take medication at the day of testing.

### Intellectual function

Two subtests from the WASI, the Vocabulary and Matrix Reasoning, were used to estimate intellectual function according to the norms presented in the test-manual [42]. The examination was performed at an outpatient clinic at the University of Bergen, where an experienced technician administrated the tests.

### Symptom scales

The Adult ADHD Self-Report Scale (ASRS) is a rating scale designed to measure current ADHD symptoms, representing the 18 DSM-IV symptoms of ADHD. The symptoms were rated on a 5-point scale (0 = never/seldom and 4 = very often), yielding a total score between 0 and 72. In this study we included the total ASRS score [43].

The Mood Disorder Questionnaire (MDQ) was included to define a subgroup with affective fluctuations [44,45]. The first 13 items are related to lifetime presence of hypomanic/manic symptoms, answered yes or no, followed by a single yes/no question about whether the symptoms have been experienced at the same time. A final question evaluates the level of impairment caused by the symptoms, rated on a four-point scale (no problem, minor problem, moderate problem, and severe problems).

The MDQ was originally designed and validated as a screening instrument for bipolar spectrum disorder [44,45]. More recent studies have been critical to this diagnostic specificity [46], and have even shown an overlap between MDQ and ADHD reported symptoms [40,47]. We therefore decided to use the more global term of affective fluctuations to describe the functionally impairing symptoms assessed by the MDQ, and strict criteria to define affective fluctuation in order to distinguish MDQ symptoms from ADHD symptoms. In the present study affective fluctuations equals a screen positive MDQ score (MDQ+), defined as: seven or more answers of "yes" on the first 13 symptom items, "yes" on the next question (co-occurrence of symptoms), and "level 3 or more" on the last question (i.e., moderate to severe impairment caused by the reported symptoms). MDQ screen negative (MDQ-) was defined as not fulfilling these criteria.

### Experimental procedure

The Attention Network Test (ANT) used in the present study is the original standard version [26], downloaded from the webpage of Jin Fan in 2005. In this version, the participants have to decide whether an arrow points to the left or right. The arrows are presented either above or below a fixation point, and may be accompanied by flankers. The test has four cue- (no cue, center, double, orienting) and three flanker conditions (congruent, incongruent, neutral). All combinations of these are randomly presented in three blocks. The calculations were based on an Excel macro downloaded from Jin Fans webpage, supplemented by measures of reaction-time, accuracy, vigilance and variability based on the calculations presented in the manual of the Conners' Continuous Performance Test, second edition [48] (Table 1). The error rates for the cue and flanker conditions included in the calculations of the attention networks were very low. The attention networks were therefore calculated from the RT measures of correct responses in the present study.

**Table 1:** Definitions of variables

*Variable*	*Definition*
Alerting network	RT for no cue - RT for double cue
Orienting network	RT for central cue - RT for orienting cue
Conflict network	RT for incongruent flanker - RT for congruent flanker
Hit reaction time	Median RT for correct responses across the test
Accuracy	Number of correct responses
Omissions	Number of omissions
SE all blocks	Standard error of RT for correct responses
Variability SE	Standard deviation of the 3 standard error values calculated for each block
RT block change	The slope of change in RT between blocks
SE block change	The slope of change in standard error of RT between blocks

The ANT was administered in a quiet test room. It was run on E-Prime software, on a stationary computer with a 17" computer screen. The participants sat at a comfortable distance from the screen and responses were collected via two input keys on the keyboard, corresponding to a left or right pointing arrow. Each administration was done individually with a research technician present in the room. The participants were asked to decide as quick as possible the direction of the middle arrow by pushing the left or right mouse button. The completion time was approximately 25 minutes.

### Statistical analysis

SPSS, version 18, was used to analyze group differences between individuals with ADHD and controls. The three network scores were included in a multivariate analysis within the GLM package, with group as a fixed factor. Univariate post-hoc tests with Tukey corrections for multiple comparisons were run to investigate group differences on each of the three networks. The analyses were repeated by including covariates, i.e., demographic variables that were significantly different between the groups. Group comparisons on the ANT measures of accuracy, variability and vigilance were investigated by using separate univariate analyses of variance within the GLM package, including covariates in case of statistically significant results. The statistical procedure was repeated within the two ADHD subgroups, by including MDQ score as a fixed binary factor. Effect sizes (d values) were calculated and interpreted according to general guidelines (*d *= 0.20 is small, *d *= 0.50 is moderate; *d *= 0.80 is large) [49].

## Results

### Description of the sample

Participants in the ADHD group were significantly older (mean age = 33.6/29.2 yrs, *t *= 2.86, *p *= .005, *d *= .53) and scored significantly lower on the test of intellectual function than the control group (mean IQ = 108.9/115.3, *t *= 2.73, *p *= .007, *d *= .52). The two groups did not differ in gender distribution. As expected, the total ASRS score was considerably higher in the ADHD than in the control group (mean ASRS = 48.7/22.4, *t *= 15.0, *p *< .001, *d *= 2.86) (Table 2).

**Table 2:** Demographic variables in the control and ADHD groups

	Controls	ADHD	ADHD MDQ+	ADHD MDQ-
	n = 56	n = 58	n = 22	n = 36
Sex	34F/22M	32F/26M	11F/11M	21F/15M
Age	29.2(7.1)	33.6(9.3)	34.2(9.3)	33.2(9.5)
IQ total score	115.3(9.6)	108.9(14.7)	103.2(14.2)	112.1(14.1)
ASRS total	22.4(9.1)	48.7(9.3)	52.3(6.2)	46.4(10.4)

Participants within the ADHD subgroups (i.e., ADHD MDQ+ and ADHD MDQ-) did not differ with respect to age (mean age = 34.2/33.2). However, the group of individuals defined as MDQ+ obtained a significantly lower total IQ (mean IQ = 103.2/112.1, *t *= 2.2, *p *= .30, *d *= .63), and higher ASRS score (52.3/46.4, *t *= 2.6, *p *= .010, *d *= .69) than the MDQ- group (Table 2). Only two participants in the control group were defined as MDQ+ (ANT results not shown).

### ANT results in the ADHD and the control group

Table 3 shows the means and standard deviations (SD) for the selected ANT variables in the control group and the ADHD group.

**Table 3:** ANT results in the control and ADHD groups

	Controls	ADHD	ADHD MDQ+	ADHD MDQ-
	n = 56	n = 58	n = 22	n = 36
Alerting network	36.0(26.2)	32.1(23.5)	22.8(28.9)	37.8(17.9)
Orienting network	42.5(25.4)	36.5(29.0)	35.4(34.3)	36.7(26.3)
Conflict network	128.2(50.3)	142.1(55.9)	162.9(54.1)	129.5(53.7)
Hit Reaction time	556.1(70.2)	576.0(92.5)	610.4(91.1)	554(88.0))
Accuracy	267.2(13.3)	258.0(12.6)	259.3(10.7)	257.4(13.3)
Ommissions	13.8(12.0)	21.0(8.8)	21.6(8.0)	20.7(9.3)
SE All Blocks	117.7(31.5)	129.1(39.2)	135.4(35.5)	125.3(41.3)
Variability SE	10.3(7.2)	13.5(8.7)	13.5(8.6)	13.5(9.2)
RT Block Change	-11.3(19.2)	-7.2(23.0)	-4.5(18.3)	-8.8(25.6)
SE Block Change	1.1(8.0)	5.1(10.5)	7.5(9.1)	3.5(11.2)

#### Attention networks

A multivariate analysis of variance (MANOVA), including the reaction-time measures of the three attention networks, showed a non-significant effect of group, Wilks' *λ *= .965, *F *= 1.3, *p *= .264. This was confirmed by the univariate analysis for the *alerting p *= .404, the *orienting p *= .244, and the *conflict network p *= .165.

#### Reaction time and accuracy measures

*Hit reaction time (RT) for correct responses *was not significantly different between the ADHD and the control group, but the *total number of hits *was significantly lower in the ADHD group than in the control group, *F *= 12.8, *p *= .001, *d *= .71. The difference remained significant after including age and intellectual function as covariates, *F *= 12.4, *p *= .001. The ADHD group committed significantly higher number of *omission errors *than the controls, *F *= 13.6, *p*< 001, *d *= .68, a difference that was retained when age and intellectual function were included as covariates, *F *= 13.4, *p *< .001.

#### Variability

The *SE of all blocks*, measuring the consistency of responses across the task, was not significantly different between the two groups. The *variability SE *score, measuring the within respondent variability throughout the task, was significantly higher in the ADHD than in the control group, *F *= 4.2, *p *= .033, *d *= .71. A trend towards statistical significance was retained when intellectual function was included as a covariate (*p *= .052), but not when age was added in the statistical model.

#### Vigilance

The *RT block change*, measuring the slope of change in reaction time throughout the test, did not differ between the ADHD and control group. The *SE block change*, measuring the slope of change in standard error of reaction time between the three blocks, was significantly higher in the ADHD than in the control group, *F *= 5.0, *p *= .027, *d *= .43. The difference was still significant when intellectual function was included as covariate, *F *= 4.4, *p *= .038, but not when adding age in the statistical model.

### ADHD subgroups: MDQ+ and MDQ-

The results for the selected ANT variables for the two ADHD subgroups are shown in the two right panels of Table 3.

#### Attention networks

A MANOVA, including the reaction-time measures of the three attention networks, showed a statistically significant difference between the two MDQ-groups, Wilks' *λ *= .836, *F *= 3.5, *p *= .021. Post-hoc tests showed that the MDQ+ subgroup obtained an overall lower score on the *alerting network *than the MDQ-subgroup, *F *= 6.0, *p *= .018, *d *= .64. On the *conflict network*, the MDQ+ subgroup obtained a significantly higher score than the MDQ- subgroup, *F *= 5.3, *p *= .026, *d *= .62, indicating that the former was more distracted by incongruent flankers than the latter. The group difference was non-significant on the *orienting network*.

A MANCOVA including the total ASRS and IQ scores as covariates was still statistically significant, Wilks' *λ *= .837, *F *= 3.3, *p *= .027. A statistical significant difference was confirmed by the univariate analyses for the *alerting network*, *F *= 7.1, *p *= .011, but not for the *conflict network*, *p *= .061.

#### Reaction time and accuracy measures

The *hit reaction time for correct responses *was significantly slower in the MDQ+ than in the MDQ-subgroup, *F *= 5.3, *p *= .025, *d *= .90. This difference was no longer statistically significant when the total IQ and ASRS scores were included as covariates (*p *= .078). The overall number of *hits across the three blocks *and the *number of omissions *were not significantly different between the two subgroups.

#### Variability and vigilance

The *variability SE*, measuring within respondent variability, the *SE block change*, measuring the slope of change in standard error of reaction time between the three blocks, and the *RT block change*, measured as the slope of change in RT throughout the task, were not significantly different between the two ADHD subgroups.

## Discussion

The present study showed that adults with ADHD did not differ from controls on ANT measures of the three attention networks, but they showed a lower accuracy, a higher intra-individual variability, and lower vigilance across the task. The effect sizes were mainly moderate, but only the accuracy measures retained statistical significance when we controlled for age and intellectual function. An important and novel aspect of the present study was the inclusion of a binary MDQ score to define an ADHD subgroup with affective fluctuations. In this subgroup we found an impact on the alerting and control networks, and on the measure of hit reaction time. The subgroup was significantly more distracted by conflicting stimuli and slower to respond. At the same time their results on the measure of the alerting network suggest that they were more alert. Furthermore, the total ASRS and IQ scores had a major impact on the reaction time and the control network in this ADHD subgroup, while the alerting network was left unaffected.

Given the original description of the test, it was somewhat surprising that adults with ADHD and controls did not differ on any measure of attention networks. According to findings in previous studies we expected to find impairment of the control network in the ADHD group [27,28,50]. Functions such as inhibition and cognitive flexibility are essential to solve cognitive conflicts as they are presented in the task. Dysfunction of these EFs are regarded as core cognitive deficits in the daily life of adults with ADHD [12,51,52], and are described as essential to understand their core symptoms [8]. In the present study, EF deficits as assessed by the conflict network were only found in the ADHD subgroup defined with affective fluctuations. The clinical importance of this finding is emphasized by the high frequency of affective symptoms among adults with ADHD [40], and that persistent affective symptoms tend to contribute to impaired function in occupational and academic areas of life [33]. Due to the lower intellectual function and higher ADHD symptom scores in the MDQ+ subgroup, our results may be explained by allocation of the individuals with the most severe ADHD to this subgroup. Although symptoms assessed by MDQ are frequently reported by individuals with ADHD [47], we argue that we used a definition of the term affective fluctuations that indicates an add on to the core ADHD symptoms: the participants should answer "yes" to the symptoms, but also confirm that the symptoms co-occurred and caused moderate to severe impairment in their daily life.

Arousal and alertness are known to be affected in ADHD [19], with a level that was expected to be different from the one shown by the control group. This was only suggested by the results in the ADHD subgroup with affective fluctuations. As anxiety symptoms are frequently found in individuals with ADHD and since anxiety symptoms are associated with high arousal, we speculate that individuals with such symptoms were mainly allocated to this ADHD subgroup. The adjustment to the new setting of ANT may have made them more prone to increased arousal. According to Eysenck, an individual with anxiety and ADHD will also be affected by a top-down influence of the EF dysfunction associated with ADHD [38]. We suggest that this may explain why both the alerting and conflict networks are affected in the ADHD subgroup defined with affective fluctuations. Such a complex interaction between the two networks has been illustrated in studies by Fan and collaborators and is supported by the fact that the alerting and conflict networks share brain networks [53,54]. MacLeod and collaborators suggested another difference between the two networks that may contribute to explain the results: the executive network is more trait-like and the alerting network more state-like [25]. It is tempting to assume that both trait and state have influenced the results in the ADHD subgroup with affective fluctuations. When we controlled for the ASRS and IQ scores, probably reflecting traits associated with ADHD, only the state-like alerting network was influenced by the affective fluctuations assessed by the binary MDQ score. However, it is important to emphasize that further studies are necessary to obtain firm conclusions about alertness in adults with ADHD and symptoms of affective disorders. The interpretations of the results in the present study should be considered with caution, not at least because the results on the alerting network are combined with slow RT in the MDQ+ subgroup.

The present study showed impairment in the ADHD group on measures of accuracy, variability and vigilance, supporting findings from earlier studies of children [29,55,56] and adults [30,35,57]. Impaired accuracy and vigilance remained when intellectual function (IQ) was included as a covariate. Earlier studies have demonstrated a more general impact of IQ on ANT measures in children with ADHD (e.g., [29]). The retained group differences in the present study indicates that results on an IQ test are not as important for ANT results in adults as they are in children. However, intellectual function did influence the results when ADHD symptoms were combined with affective symptoms, illustrating the complex relation between core and comorbid symptoms of ADHD, EF and intellectual function.

The present study has limitations related to the diagnosis of ADHD and the definition of affective fluctuations. The adults with ADHD included in the study were evaluated by several clinicians before inclusion in the study, probably yielding a large heterogeneity within the ADHD group. On the other hand, our ADHD sample probably represents adults with an ADHD diagnosis as encountered in a clinical setting. Affective fluctuations were assessed according to a self-report questionnaire, originally designed to measure symptoms of bipolar spectrum disorders. However, it has been shown that most adults with ADHD with a positive MDQ score do not fulfill criteria for this disorder [40], and that a positive MDQ score may be found in a range of psychiatric diagnoses [47]. The conclusions of the present study will therefore not be restricted to a specific comorbidity group. It is also a limitation that the adults with ADHD were only asked not to take medication at the day of testing. Although we thus do not know if the wash-out of the medication was complete, our results are supported by the fact that methylphenidate use was more frequently reported in the MDQ- than the MDQ+ group. Finally, the study did not include biomarkers of the attention networks. Previous studies suggest that data derived from imaging techniques may be more sensitive than behavioral data collected in a standard test condition outside the scanner [27,28,50]. To extend the exploration of competition between the neural substrates of the alerting and conflict networks to neuroimaging of the here described subgroups would therefore be an important asset.

## Conclusions

The present study suggests that adults with ADHD are less accurate, have a higher level of variability and a lower vigilance than adults without ADHD, and that affective fluctuations make adults with ADHD more alert, but slower and more distracted by conflicting stimuli. Our results indicate that the cognitive heterogeneity among adults with ADHD at least partly is explained by affective symptoms. By these results the present study emphasizes the importance of characterizing and taking these symptoms into account in research and clinical work with adults with ADHD.

## Competing interests

The authors declare that they have no competing interests.

## Authors' contributions

AJL: Design, statistical analyses and writing of the manuscript.

SA: Data analysis, comments on the manuscript.

HH: Cognitive assessment, comments on the manuscript

KP: Comments on the manuscript.

AH: Comments on the manuscript.

JH: Head of the national ADHD project, comments on the manuscript.

All co-authors have read and accepted the final version of the manuscript.
